# The Mechanisms Leading to Distinct Responses to PD-1/PD-L1 Blockades in Colorectal Cancers With Different MSI Statuses

**DOI:** 10.3389/fonc.2021.573547

**Published:** 2021-03-05

**Authors:** Guanglin Cui

**Affiliations:** ^1^ Research Group of Gastrointestinal Diseases, The Second Affiliated Hospital of Zhengzhou University, Zhengzhou, China; ^2^ Faculty of Health Science, Nord University, Bodø, Norway

**Keywords:** therapy, inhibitor, immune checkpoint, metastasis, colorectal cancer

## Abstract

Current clinical studies showed distinct therapeutic outcomes, in which CRC patients with mismatch repair-deficient (dMMR)/microsatellite instability high (MSI-H) seem to be relatively more “sensitive” in response to anti-programmed death-1 receptor (PD-1)/programmed death-1 receptor ligand 1 (PD-L1) therapy than those with mismatch repair-proficient (pMMR)/microsatellite instability-low (MSI-L). The mechanisms by which the same PD-1/PD-L1 blockades lead to two distinct therapeutic responses in CRC patients with different MSI statuses remain poorly understood and become a topic of great interest in both basic research and clinical practice. In this review of the potential mechanisms for the distinct response to PD-1/PD-L1 blockades between dMMR/MSI-H CRCs and pMMR/MSI-L CRCs, relevant references were electronically searched and collected from databases PubMed, MEDLINE, and Google scholar. Sixty-eight articles with full text and 10 articles by reference-cross search were included for final analysis after eligibility selection according to the guidelines of PRISMA. Analysis revealed that multiple factors *e.g.* tumor mutation burden, immune cell densities and types in the tumor microenvironment, expression levels of PD-1/PD-L1 and cytokines are potential determinants of such distinct response to PD-1/PD-L1 blockades in CRC patients with different MSI statuses which might help clinicians to select candidates for anti-PD-1/PD-L1 therapy and improve therapeutic response in patients with CRC.

## Introduction

Colorectal cancer (CRC) is the third most common type of cancers and the second leading cause of cancer-related death in developed countries. Although numerous attempts have been made to increase the overall survival rate of CRC patients, the improved prognosis in CRCs is still heavily dependent on early diagnosis and complete resection of the primary tumor. Unfortunately, metastasis is often observed at the time of diagnosis, and curative surgical resection becomes impossible in these patients. Thus, to improve the prognosis of metastatic CRC (mCRC) patients, many researchers have investigated the escape immunosurveillance mechanisms by which the CRC progresses and metastases persistently (1–4). A growing body of evidence from various studies has demonstrated that immune checkpoints are negative regulators for host anti-tumor immune function and could significantly suppress the host anti-tumor immune reactivity (5–7). Therefore, one of the proposed mechanisms for a tumor’s ability to escape immunosurveillance is by targeting of immune checkpoint molecules and becomes an important therapeutic strategy for the treatment of human cancers. Indeed, recent studies revealed that immune checkpoint blockades with monoclonal antibodies (mAbs) that target the programmed cell death receptor 1 (PD-1) and its ligand (PD-L1) have been widely used in treating human cancers and showing in an enhanced host anti-tumor immunity and increase survival rates in a variety of malignancies including melanoma (8), renal cell carcinoma (9), and non-small cell lung cancer (10). The therapeutic efficacy of anti-PD-1/PD-L1 mAbs has been evaluated in patients with solid tumors including mCRC (11–14). However, unlike the efficacy reported in patients with melanoma, the therapeutic response of anti-PD-1/PD-L1 mAbs in patients with mCRC is very different between mismatch repair-deficient (dMMR)/microsatellite-instability-high (MSI-H) tumors and mismatch repair-proficient (pMMR)/microsatellite-instability-low (MSI-L) or microsatellite-stability (MSS) tumors (11, 15–18). Results of clinical studies or trials showed that CRC patients with dMMR/MSI-H seem to be relatively more “sensitive” in response to anti-PD-1/PD-L1 mAbs than those CRCs with pMMR/MSI-L: dMMR/MSI-H CRCs treated with anti-PD-1/PD-L1 mAbs exhibit significantly enhanced sustained clinical responses. However, anti-PD-1/PD-L1 therapies are ineffective in CRC patients with pMMR/MSI-L (12, 13, 19). The mechanisms leading to distinct therapeutic outcomes between dMMR/MSI-H CRCs and pMMR/MSI-L CRCs have been a topic of great clinical interest and studied.

Therefore, this manuscript reviews the current understanding of the potential mechanisms leading to a distinct therapeutic response to anti-PD-1/PD-L1 mAbs between dMMR/MSI-H CRCs and pMMR/MSI-L CRCs.

## Search Strategy and Selection Criteria

### Literature Search Strategy

**Figure 1:**
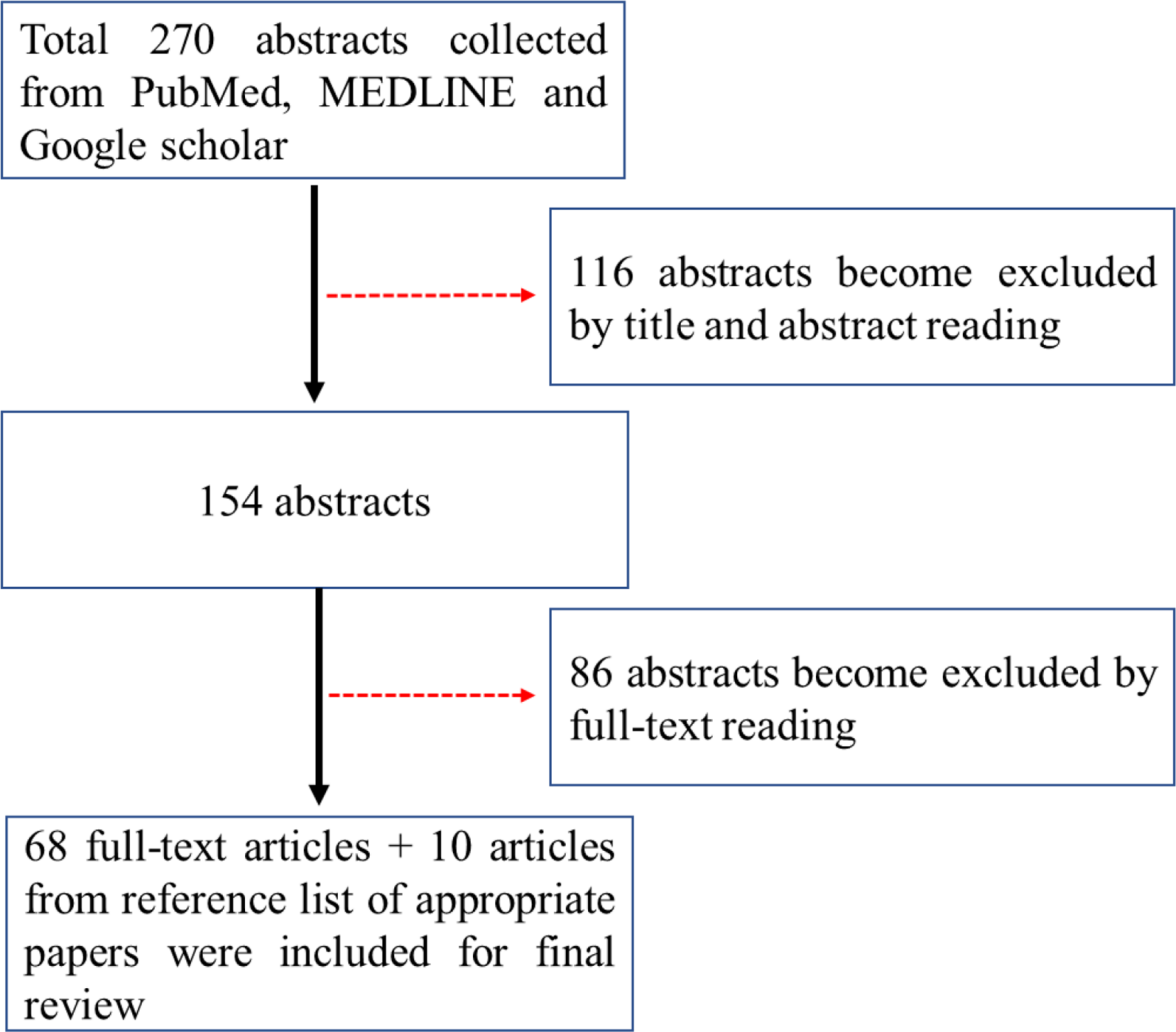
Prisma flow chart for the search of the literatures.

Relevant electronic literature search was conducted in academic databases PubMed, MEDLINE and Google scholar by the author using the search terms “anti-PD-1”, “anti-PD-L1”, “immune checkpoint inhibitor”, “response”, “predicators”, “colorectal cancer”, “metastasis”, “dMMR”, “pMMR” and “MSI” from inception to September 2020. Of 270 abstracts hit, sixty-eight articles with full text were included for final analysis according to the guidelines of Preferred Reporting Items for Systematic Reviews and Meta-Analyses (PRISMA) (see **Figure 1**). Ten articles selected from reference lists of appropriate papers as an additional literature source were included.

### Inclusion and Exclusion Criteria

The following selection criteria were used: (1) articles published in English; (2) full-text available; (3) studies or trials conducted in human CRCs; (4) relevant mechanistic studies conducted in mice; (5) articles only published as abstract or non-full text publications (case reports, editorials, letters to editors, or meeting abstracts) were excluded; (six) studies were rejected if they lacked sufficient information to study anti-PD-1/PD-L1 therapies in CRC.

The following information was extracted: year of publication; country of origin; study design; journal; study population size; and mean age of patients. A meta-analysis was not carried out because of the heterogeneity, differences in metrics and settings in the included publications.

## A Brief Overview of the Role of PD-1/PD-L1 in the Escape Immunosurveillance of CRC

PD-1 is an inhibitory coreceptor that is highly expressed on the cell surface of various types of immune cells such as T and B lymphocytes and natural killer (NK) cells (20, 21). In the tumor microenvironment, PD-1 is also expressed in tumor-infiltrating lymphocytes (TILs) and participates in the modulation of host anti-tumor immune response (20). The modulation effect of PD-1/PD-L1 on immune reactivity has been well documented by intensive studies. Physiology, the binding of PD-1 with its ligand PD-L1 leads to an inhibitory efficiency of T cell activation and suppression of cytokine productions, *i.e.*, interferon-*γ* (IFN-*γ*), tumor necrosis factor-α (TNF-α) and interleukin (IL)-2 from immune cells (22). These effects hamper the overreaction of immune response and help to maintain immune homeostasis. In the tumor microenvironment, PD-L1 is expressed on the tumor cell surface and the binding of PD-1 expressed by TILs may lead to the functional inactivation of TILs and loss of their ability to kill tumor cells that result in immune resistance in patients with tumors (23, 24). Therefore, the PD-1/PD-L1 pathway has been recognized as a negative modulator of immune response by restricting the function of TILs in the tumor immune microenvironment (TIME), and inhibition of the PD-1/PD-L1 pathway by administration of mAbs can reactivate the function of cytotoxic T lymphocytes (CTLs) and their ability to attack tumor cells (25).

In addition to anti-PD-1/PD-L1 mAbs, blockades of other immune checkpoint factors such as cytotoxic T-lymphocyte associated protein 4 (CTLA4) mAbs have been shown to induce significantly enhanced T cell proliferation and IL-2 production (26, 27) and decreased density of regulatory T cells (Tregs) in the TIME (27, 28), which relates to an improved prognosis in patients with tumors (7). Taken together, current available scientific evidence suggests that the immune checkpoint PD-1/PD-L1 pathway plays a critical role in the process of immunoediting and tumor progression and metastasis (29). Pharmacologically, PD-1/PD-L1 pathway signals can be easily targeted. Recent immunotherapeutic strategies based on mAb targeting of immune checkpoint molecules such as PD-1 and its ligand PD-L1 and CTLA4 have shown a promising therapeutic response that leads to increase the functional activation of immune cells and enhanced host anti-tumor immune response in patients with tumors such as malignant melanoma (8, 25), renal cell cancer (30), lung cancer (30) and CRC (7).

## Distinct Therapeutic Efficacy of Anti-PD-1/PD-L1 mAbs Between Patients With dMMR/MSI-H CRC and Patients With pMMR/MSI-L or MSS CRC

According to its etiology, CRCs can be divided into sporadic, familial, or hereditary (30). Approximately 70–75% of CRCs are sporadic CRCs and become the main type of CRCs in the clinic. Clinical studies demonstrated that dMMR/MSI-H status is observed in approximately 15–20% of sporadic CRCs (31, 32); higher immune cell infiltration and better prognosis are more frequently reported in dMMR/MSI-H tumors than pMMR/MSI-L tumors (33). The therapeutic efficacy of anti-PD-1 and anti-PD-L1 mAbs in patients with CRCs has been recently evaluated. A distinct therapeutic efficacy of anti-PD-1/PD-L1 mAbs between dMMR/MSI-H tumors and pMMR/MSI-L tumors was demonstrated in numerous clinical studies (15, 16, 30, 34–41). Overman et al. (36) investigated the efficacy and safety of nivolumab (an anti-PD1 mAb) combined with ipilimumab (anti-CTLA-4 mAb, another immune checkpoint inhibitor) in patients with MSI-H mCRC *vs*. non-MSI-H mCRC. They reported that nivolumab as a single bioagent or in combination with ipilimumab could result in 31% disease control in mCRC patients with MSI-H and 10% disease control in patients with MSS mCRC (36). Interestingly, their results revealed that a high tumor mutational burden (TMB) in mCRC may predict a better response, and nivolumab in combination with ipilimumab is a promising new therapeutic strategy for dMMR/MSI-H mCRC (36). Le et al. (42) also reported similar findings in CRC patients with different MSI statuses. The immune-related objective response rate (ORR) and the immune-related progression-free survival (PFS) rate to anti-PD-1 mAbs at primary endpoints of the study (20 weeks) were remarkedly higher in dMMR CRCs than that in pMMR CRC. Taken together, current studies with anti-PD-1 mAbs suggested that the therapeutic efficacy is generally unencouraging and response rates in patients with pMMR/MSI-L mCRC were seen only rarely. Thus, the dMMR status is an important factor in influencing therapeutic response of anti-PD-1 mAbs in treating patients with CRCs (43). Several reviews have summarized the distinct therapeutic response of anti-PD-1/PD-L1 mAbs in CRC patients with different MSI statuses; readers can refer to these articles.

In addition to anti-PD-1 mAbs, the therapeutic efficacy of anti-PD-L1 mAbs in mCRCs is also being evaluated. Brahmer et al. assessed the efficacy of an anti-human PD-L1 mAb in a cohort of more than 200 solid tumors that included 18 CRC patients. The results showed that none response was seen in patients with CRC (44). Furthermore, unlike its therapeutic efficacy demonstrated in other types of solid tumors such as advanced non-small cell lung cancer (45) and metastatic urothelial cancer (46), the response rate of atezolizumab (a humanized anti-PD-L1 mAb) in MSS CRCs is not encouraging (47). The therapeutic analysis of the combination of cobimetinib (a MEK protein kinase mAb) with atezolizumab in 23 patients with mCRC suggests that the response rate was very low in CRC patients with pMMR/MSI-L (47).

For details, readers can refer to published literatures; several reviews have summarized such distinct therapeutic response of anti-PD-1/PD-L1 mAbs between dMMR/MSI-H CRCs and pMMR/MSI-L CRCs (7, 15, 16, 18, 48–51). Further studies or trials should focus on how to improve therapeutic response rate of anti-PD-1/PD-L1 mAbs in patients with pMMR/MSI-L CRCs.

## The Possible Mechanisms Leading to a Distinct Therapeutic Efficacy of Anti-PD-1/PD-L1 mAbs Between dMMR/MSI-H CRCs and pMMR/MSI-L CRCs

Clinical studies and trials have shown that patients with dMMR/MSI-H CRC and pMMR/MSI-L or MSS CRC respond inconsistently to anti-PD-1/PD-L1 mAbs, which is associated with differences in the components of TIME. Indeed, it has been shown that multiple factors existed in the TIME, including immune cell phenotypes, cytokine networks, and immune checkpoints, differ between dMMR/MSI-H CRCs and pMMR/MSI-L CRCs (12, 15, 52–57) (see summarization in **Table 1**).

**Table 1:** Difference of tumor immune microenvironment (TIME) between dMMR/MSI-H CRCs and pMMR/MSI-L CRCs.

Components (reference ID)	dMMR/MSI-H *vs*. pMMR/MSI-L
TIL (43)	>
Effector-memory T cells (58)	>
TH1 cells (58)	>
Macrophage (59)	>
DC (59)	>
Foxp3 low non-suppressive Tregs (60)	>
PD1/PD-L1 expression (58, 61, 62)	>
CTLA4 expression (5)	>
IFN-γ expression (25, 63, 64)	>
IDO (65, 66)	>

CTLA4, Cytotoxic T-lymphocyte-associated antigen 4, DC, dendritic cell; IDO, indolamine 2′3′-dioxygenase; IFN-γ, interferon-γ; PD-1, programmed death-1 receptor; PD-1L, programmed death-1 receptor ligand; MSI-H, microsatellite-instability-high; MSI-L, microsatellite-instability-low; dMMR, mismatch repair-deficient; pMMR, mismatch repair-proficient; TIL, tumor infiltrating lymphocyte; Treg, regulatory T cell.

Studies have revealed that TMB in dMMR/MSI-H CRCs is significantly higher than that in pMMR/MSI-L CRCs (48, 67–69), and an increased amount of TMB in dMMR/MSI-H tumours can result in a 20-time higher rate of mutation and a stronger immune response than pMMR/MSI-L tumours (52, 53, 58), which are reflected in the very different composition and function of immune infiltrates in the TIME (70–73). Therefore, TMB appears to be an important biomarker for patients with dMMR/MSI-H CRC in response to anti-PD-1 therapy (68, 74–77). Schrock et al. reported that TMB was strongly associated with objective response and favorable progression-free survival, by univariate (P < 0.001) and multivariate analysis in MSI-H mCRC patients treated with anti-PD-1/PD-L1 mAbs (68). Fotios Loupakis et al. reported that higher TMB and increased number of TILs in patients with dMMR/MSI-H CRCs were likely related to a better response to therapies of checkpoint inhibitors (78). Consequently, numerous studies demonstrated that increased densities of infiltrating immune cells *i.e.* TH9 subset may possibly contribute to the improved therapeutic response of dMMR/MSI-H CRCs to anti-PD-1/PD-L1 mAb treatment (59, 78–80). Zhang et al. (54) have immunohistochemically shown that the TIME of dMMR/MSI-H CRCs differs from that of pMMR/MSI-L CRCs. The density of PD-1 positive TILs was higher in CRC patients with dMMR/MSI-H than those with pMMR/MSI-L. The positive rate of PD-L1 on immune cells in the TIME also differed between dMMR/MSI-H CRCs and pMMR/MSI-L CRCs. In addition, the level of immunosuppressive factor indoleamine 2,3 dioxygenase (IDO) expression was greater in CRC patients with dMMR/MSI-H CRCs than those with pMMR/MSI-L (65, 66).

PD-L1 is the ligand for PD-1 and plays an important negative regulatory effect by binding PD-1, resulting in the restricting of anti-tumor function of T lymphocytes (81, 82). Liu et al. (43) demonstrated that dMMR/MSI-H CRCs more frequently exhibited higher populations of TILs and PD-L1-positive cells than pMMR/MSI-L CRCs. Furthermore, PD-L1 expression in patients with CRC was associated with clinicopathologic and molecular features and a worse outcome in CRC patients with dMMR/MSI-H than those with pMMR/MSI-L (83). PD-L1 expression was closely related to dMMR/MSI-H status in patients with CRC. Taube et al. reported that PD-L1 expression was identified in 42.4% of dMMR/MSI-H CRCs with TILs and only 18.0% pMMR/MSI-L CRCs with TILs (43). They also found that tumor PD-L1 expression reflected an immune-active microenvironment, while it did so in association with other immunosuppressive molecules, including PD-1 and PD-L2, it was the single factor most closely correlated with response to anti-PD-1 mAbs in patients with melanoma, non-small cell lung carcinoma, renal cell carcinoma, CRC, or castration-resistant prostate cancer (57). Liu et al. (84) immunohistochemically examined the cellular localization of PD-L1 in 73 dMMR CRCs and 56 pMMR CRCs respectively. They found that the expression of PD-L1 was primarily observed in tumor infiltrating immune cells, particularly in immune cells at the site of invasive front with tumor–stroma-interface. The population level of immune cell positive for PD-L1 staining was significantly higher in dMMR tumors than in pMMR tumors (84). Both Gatalica and Inaguma have confirmed a similar result in CRC patients with different MSI statuses (61, 62). dMMR/MSI-H CRC patients with a higher PD-L1 level had a deeper functional suppression of TILs than those pMMR/MSI-L CRC patients with a lower PD-L1 level, and blocking PD-1/PD-L1 might relieve such suppression and reactivate the function of TILs (61, 62). Therefore, PD-L1 expression in diverse solid tumors might stratify patients in terms of response to anti-PD-1/PD-L1 immunotherapy and sensitivity to anti-PD-1/anti-PD-L1 mAbs (85), and PD-L1 expression was related to intrinsic and adaptive immune resistance in cancer patients receiving immunotherapy (86). Analysis of TCGA data has also revealed that the mRNA expression levels of both PD-L1 and PD-L2 were significantly upregulated in dMMR/MSI-H CRCs compared with pMMR/MSI-L CRCs (87). Indeed, clinical studies identified that mCRC patients with higher PD-L1 expression in CRC cells might had a higher response rate than those patients with lower PD-L1 expression in CRC cells (42, 88), which was also supported by a meta-analysis of PD-L1 expression in patients with melanoma and lung and genitourinary cancers (89). However, Le Flahec et al.’s (90) immunohistochemistry compared PD-L1 expression in whole tumor specimens and tissue microarray slides between dMMR/MSI-H CRCs and pMMR/MSI-L CRCs. They reported no significant difference in PD-L1 expression levels between dMMR and pMMR CRCs. Thus, they concluded that the value of PD-L1 in explaining the different therapeutic response rate between dMMR CRCs and pMMR CRCs remains unclear, and further studies are required.

Taken together, PD-1 expression observed on the increased populations of immune cells in dMMR/MSI-H CRCs may act as a modulatory factor for the immune response. The use of anti-PD-1 mAbs blocks the expression of PD-1 on tumor cells and TILs and could further reactivate other types of immune cells and finally enhances host anti-tumor immune response in patients with dMMR/MSI-H CRC. Therefore, the response rate to anti-PD-1 and anti-PD-L1 mAbs in patients with dMMR/MSI-H CRC is higher than that in patients with pMMR/MSI-L mCRC.

Different levels of cytokines produced by both immune cells and tumor cells in the tumor microenvironment may also contribute to a distinct therapeutic response between dMMR/MSI-H CRCs and pMMR/MSI-L CRCs. For example, IFN-*γ* produced by activated T lymphocytes is an important modulator of PD-L1 expression, and it can significantly upregulate PD-L1 expression in both tumor cells and immune cells in the TIME (69, 86). Studies have shown that responders had elevated expression levels of IFN-*γ* and IFN-*γ*-inducible genes prior anti-PD-L1 mAb treatment in patients with melanoma or renal cell carcinoma (25, 63, 64). However, its role in influencing the response rate to anti-PD-1/PD-L1 mAb therapy in patients with dMMR/MSI-H CRC needs further investigation.

Finally, host anti-tumor immunity is regulated by a complex immune cellular network in patents with CRC (1, 3, 70, 91–93). For instance, a number of recent studies have reported that diverse TH subsets TH9, TH17 and TH22 and their main cytokines such as IL-9, IL-17 and IL-22 participate in the modulation of immune response in patients with CRC (91). Immune scores between dMMR/MSI-H CRCs and pMMR/MSI-L or MSS CRCs were also very different (94, 95) in which, populations of CD3-positive and CD4-positive TILs in tissue microarray samples collected from the tumor center and invasive front were significantly higher in CRC patients with dMMR/MSI-H than those with pMMR/MSI-L (94). The phenotypes of infiltrating immune cells were also different between dMMR/MSI-H and pMMR/MSI-L CRCs (95). In addition to the infiltration of lymphocytes, many other cellular components of the CRC TIME might be different (96). For example, local immunosuppressive cells, including Tregs and myeloid-derived suppressor cells (MDSCs), were ordinarily observed in the CRC TIME (97, 98). Le Gouvello et al. reported remarkable increased levels of Foxp3-positive Tregs and IL-17 in CRC patients with pMMR/MSI-L (99). These cells might be involved in the determination of anti-PD-1 therapeutic efficacy in patients with CRC. One study conducted in the human hepatoma cell lines showed that Tregs potentially inhibited IFN-*γ* secretion and the cytotoxicity of CD8-positive T cells (100). Moreover, Bauer K et al. (101) reported that the existence of Tregs greatly influenced the frequency of effector T cells in response to specific MSI-H-related frameshift peptides in CRC. Dyck and colleagues (102) reported that high PD-1 expression correlated with increased tumor-infiltrating Tregs, and reduced effector T cells and blocking PD-1 might effectively enhance antitumor immunity. Zhang et al. (103) have further shown that PD-1 mAbs could reverse Treg suppression. Toor et al. (21) demonstrated that Tregs might hamper the response to immune checkpoint blockade (anti-PD-1 mAb) in patients with CRC, and the administration of an anti-PD-1 mAb in mice could significantly decrease tumor-infiltrating Tregs and increase TILs in the CRC TIME (104). Interestingly, a higher expression of FoxP3 (a marker of Tregs) has been demonstrated in patients with pMMR/MSI-L CRC than in those with dMMR/MSI-H CRC, indicating a deeper degree of immunosuppression in these patients. As a result, a higher proportion of Tregs is associated with a poor prognosis for immunotherapy, and the rate of therapeutic response to anti-PD-1 mAbs might be lower in patients with pMMR than that in patients with dMMR. Therefore, eliciting an enhanced response to anti-PD-1/PD-L1 mAbs in patients with pMMR/MSI-L CRC may require combined therapeutics with other anti-immunosuppressive, antiangiogenic or anti-immune checkpoint bioagents (42, 105).

**Figure 2:**
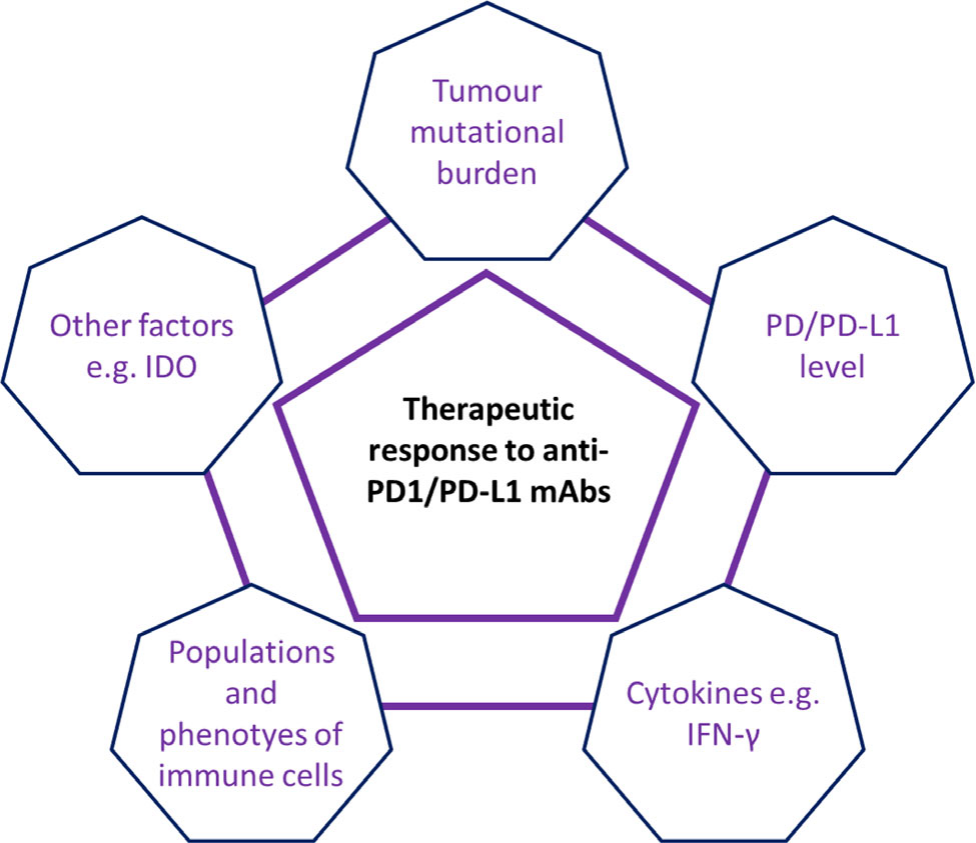
Schematic summary of multiple factors involving in the anti-PD1/PD-L1 therapeutic response in patients with CRC. Current evidence has suggested that multiple factors *e.g.* tumor mutational burden (TMB), IFN-*γ* level, populations and phenotypes of immune cells and other factors (*e.g.* immunosuppressive factors IDO) were involved in the regulation of anti-PD1/PD-L1 therapeutic response in patients with CRC.

Thus, accumulating evidence suggests that multiple factors are involved in the lack of sensitivity to anti-PD1/PD-L1 mAb therapy seen in patients with pMMR/MSI-L or MSS tumours (see **Figure 2**). The above possible mechanisms might provide useful information for future improvements. For example, the host immune response to TMB was very different between patients with dMMR/MSI-H CRC and those with pMMR/MSI-L CRC (76, 94), and a single anti-PD-1/PD-L1 mAbs could not block all the regulatory pathways/signals. Recent studies revealed that the calcium/calmodulin-dependent protein kinase 1D (CAMK1D) and the m6A demethylase Alkbh5 may regulate the tumor cells refractory to anti-PD-L1 treatment (106, 107). However, the difference of the CAMK1D and m6A demethylase Alkbh5 expressions between dMMR/MSI-H and pMMR/MSI-L CRCs has not been well studied so far. Furthermore, a recent study suggested that transcriptional factor STAT was involved in the response to anti-PD-1 in patients with MSS CRCs (108). Finally, it is important to state that not all dMMR/MSI-H CRCs respond to immunotherapy. Combinations of anti-PD-1/PD-L1 mAbs with other anti-tumor agents may improve the therapeutic efficacy in patients with CRCs (69). Trials of atezolizumab in combination with both bevacizumab and cobimetinib mAbs have opened the way for combination strategies, which could extend the indication for immune checkpoint inhibitors to pMMR mCRC. Similarly, the combination of nivolumab and ipilimumab mAbs in dMMR/MSI-H mCRC patients has shown an encouraging clinical outcome (36).

## Translational Significance of Mechanisms Leading to Distinct Outcomes Between dMMR/MSI-H CRCs and pMMR/MSI-L CRCs

Accumulative evidence has suggested that multiple factors such as TMB, expression levels of PD-1/PD-L1 and cytokines and immune infiltrates are involved in the mechanisms of distinct response to anti-PD-1/PD-L1 mAbs between dMMR/MSI-H CRCs and pMMR/MSI-L CRCs. The discovery of potential mechanisms leading to distinct therapeutic outcomes of anti-PD-1/PD-L1 therapy seen between dMMR/MSI-H CRCs and pMMR/MSI-L CRCs might have an important translational significance (55, 109).

Firstly, the study of potential mechanisms leading to a distinct therapeutic efficacy of PD-1/PD-L1 inhibitors seen between dMMR/MSI-H and pMMR/MSI-L CRCs might help clinicians to select candidates for anti-PD-1/PD-L1 therapy. Anti-PD-1/PD-L1 therapy is an expensive biotherapeutic. Treating cost studies have revealed that treating with immune checkpoint inhibitors may averagely cost $1 million per patient (110). By analyzing relative factors, clinicians can identify candidates (CRC patients) who will benefit from anti-PD-1/PD-L1 therapy. For example, studies have reported that mCRC patients with higher PD-L1 expression might have a higher response rate than those patients with lower PD-L1 expression (42, 88). Furthermore, it may help clinicians to select reliable biomarker in the evaluation of anti-PD-1/PD-L1 therapeutic response in patients with CRC. Clinicians can measure the expression level of PD-L1 in tumor cells and immune cells examined by diverse techniques *i.e.* immunohistochemistry and *in situ* hybridization, and in whole tissue specimens measured by real-time PCR to provide important predicating information of response prior treatments. To improve and optimize the efficacy of anti-immune checkpoint therapies, a combination of anti-PD-1/PD-L mAbs with other anti-tumor bioagents probably being the most relevant strategy (36, 111, 112). For instance, several studies showed that IFN-*γ* can significantly upregulate PD-L1 expression in both tumor cells and immune cells (69, 86, 113). This finding raises an interesting possibility that whether combination biotherapy of anti-PD-1/PD-L1 mAbs with IFN-*γ* can induce an enhanced response rate in patients with CRC should be tested. Moreover, an improved therapeutic efficacy has been demonstrated in cancer-favoring oncolytic vaccinia virus combined with anti-PD-1 in treating mouse CRC (106). Cai et al. (114) reported an upregulated effect of angiogenesis inhibitor apatinib on the expression of PD-1 at mRNA and protein levels but a downregulated effect on IFN-*γ* secretion from T cells in various colon cancer cells. The combination of apatinib with anti-PD-1 antibody could result in a better efficacy in a syngeneic mouse model (CT-26 cells in Balb/c) than single-drug treatment. More recently, Knudson et al. showed that IL-15 super-agonist N-803 plus anti-PD-L1 mAb could reduce MC38-CEA (colon cancer cell) tumor burden and increased survival rate as compared to N-803 and anti-PD-L1 monotherapies in MC38-CEA colon tumor-bearing mice (115). An enhanced therapeutic effect of F8-IL2 (an antibody-IL2 fusion protein) bombinated with anti-PD-1, anti-PD-L1 and anti-CTLA-4 antibodies was observed in immunocompetent mice bearing CT26 tumours (116). These studies may provide a rationale for the combination of anti-PD-1 antibody with other anti-tumor agents for CRC treatment in the clinic. Finally, as the mechanisms responsible for distinct responses to PD-1/PD-L1 mAbs in patients with CRC are elucidated, how to convert a ‘nonimmunogenic’ CRC into an ‘immunogenic’ CRC becomes a critical important issue (18, 117). Wang et al. recently investigated the efficacy of fruquintinib (a novel anti-vascular endothelial growth factor receptor tyrosine kinase inhibitor) plus anti-PD-1 mAb for MSS CRC in a murine syngeneic model of CT26 cells and verified that cotreatment significantly inhibited tumor growth and promoted survival time for tumor-bearing mice compared with the single drug alone (105).

## Concluding Remarks

**Figure 3:**
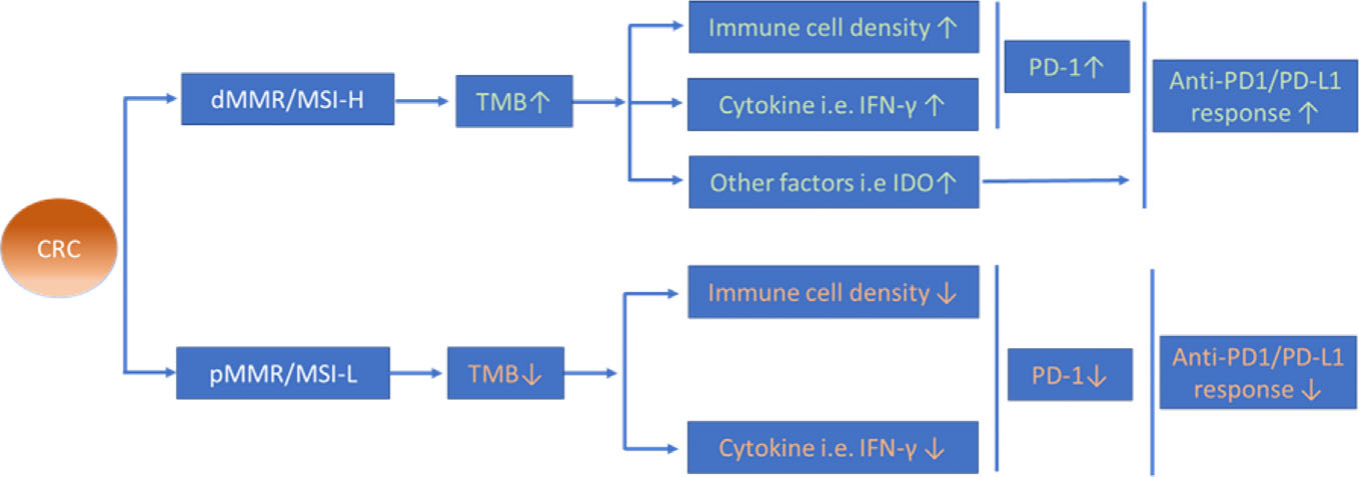
Possible mechanisms of different anti-PD-1/PD-L1 therapeutic efficacy between CRC with dMMR/MSI-H and pMMR/MSI-L. Increased TMB in dMMR/MSI-H CRCs is proposed to induce higher infiltration of immune cells and expression level of cytokines in the tumour microenvironment that might contribute to the distinct therapeutic efficacy of anti-PD-1/PD-L1 therapy between dMMR/MSI-H CRCs and pMMR/MSI-L CRCs.

Division of mCRCs into dMMR/MSI-H and pMMR/MSI-L subsets yields a distinct therapeutic response to anti-PD-1/PD-L1 immunotherapies, in which dMMR/MSI-H CRCs seem to be relatively more “sensitive” in response to anti-PD-1/PD-L1 mAbs than those CRCs with pMMR/MSI-L. The possible mechanisms leading to such distinct outcomes may relate to multiple factors *e.g.* TMB, TILs, and immunosuppressive cells, the expression level of PD-L1 and the complex cytokine network in the CRC microenvironment (see **Figure 3**). Future studies should discover strategies that determine how to convert a ‘non-immunogenic’ CRC into an ‘immunogenic’ CRC and improve the therapeutic response rate to anti-PD-1/PD-L1 mAbs in patients with pMMR/MSI-L mCRC and the optimal strategy for identifying CRC patients who will benefit from anti-PD-1/PD-L1 mAb therapy prior treatments in the clinic.

## Author Contributions

The author confirms being the sole contributor of this work and has approved it for publication.

## Funding

This study was supported by the National Nature Science Foundation of China (Program No. 81071969) and the Medical Research Program, Northern Norway Regional Health Authority, Norway (Program No. SFP-44-04).

## Conflict of Interest

The author declares that the research was conducted in the absence of any commercial or financial relationships that could be construed as a potential conflict of interest.

## Abbreviations

APC, antigen-presenting cells; PRISMA, Preferred reporting items for systematic reviews and meta-analyses; CAMK1D, the calcium/calmodulin-dependent protein kinase 1D; CRC, colorectal cancer; CTL, cytotoxic T lymphocyte; CTLA4, cytotoxic T-lymphocyte associated protein 4; dMMR, mismatch repair-deficient; IDO, indolamine 2′3′-dioxygenase; IFN-*γ*, interferon-*γ;* IL. Interleukin; mAb, monoclonal antibody; mCRC, metastatic CRC; MDSCs, myeloid-derived suppressor cells; MHC, major histocompatibility complex; MSI-H, microsatellite-instability-high; MSI-L, microsatellite-instability-low; NK, natural killer; ORR, the objective response rate; OS, overall survival; PD-1, programmed death-1 receptor; PD-L1, programmed death-1 receptor ligand; PFS, progression-free survival; pMMR, mismatch repair-proficient; TGF-*β*, transforming growth factor-*β;* TIL, tumor infiltrating lymphocyte; TIME, tumor immune microenvironment; TMB, tumor mutational burden; TNF-α, tumor necrosis factor-α; Treg, regulatory T cell.
